# Knowledge, attitudes, and practices regarding cardiovascular disease prevention among middle school students in China: a cross-sectional study

**DOI:** 10.3389/fpubh.2024.1301829

**Published:** 2024-01-26

**Authors:** Xin Yang, Qiang Qin, Yifei Wang, Zhaopeng Ma, Qiurong Li, Fusheng Zhang, Yanbai Han, Hongli Wang

**Affiliations:** ^1^College of Physical Education and Health, Guangxi Normal University, Guilin, China; ^2^Department of Physical Education, Guilin Medical University, Guilin, China

**Keywords:** cardiovascular disease, adolescents, knowledge, attitude, preventive practice

## Abstract

**Background:**

The prevalence of cardiovascular disease (CVD) is rapidly increasing globally. With a concerning increase among adolescents due to unhealthy habits, obesity, and hypertension, understanding the current status of knowledge, attitudes, and practices (KAP) related to CVD prevention among middle school students is crucial for developing effective school-based health programs to prevent CVD.

**Methods:**

The analytic cross-sectional survey is used in questionnaires to assess KAP related to CVD prevention among middle school students (*N* = 17,731) from 50 schools across 16 provinces in China in June–July 2023. The pass rate of KAP scores is categorized as good and poor. Independent predictors of good KAP of CVD prevention are ascertained using a binary logistic regression model.

**Results:**

The study surveyed 8,118 (45.78%) junior high school students and 9,613 (54.22%) high school students. The overall mean [standard deviation (SD)] for the knowledge, attitude, and practice scores were 26.88 (8.12), 53.53 (7.22), and 39.80 (5.96), respectively. The knowledge scores had the lowest pass rate at 56.89%. Only 6.83% of the students know “the definition of blood pressure in adolescents.” Attitudes toward health were positive, though the attitude regarding “the danger of prolonged sedentary to cardiovascular health” scored lowest at 73.55%. The practice section had a pass rate of 89.30%; 40.27% of students reported that they spend more than an hour a day on screens. Only one-third of the students would go to bed before 12 o’clock. In univariate analysis, junior high school and high school students differed significantly in knowledge and practice (*p* < 0.001), but attitude did not differ significantly (*p* = 0.103).

**Conclusion:**

The majority of students lack sufficient knowledge about CVD. It is also found that socioeconomic background, family environment, and educational levels have an impact on cardiovascular health behaviors among students. Strengthening health education involving students, parents, teachers, and communities is essential to promote health knowledge and practices among adolescents.

## 1 Introduction

Cardiovascular disease (CVD) is one of the major non-communicable diseases with a significant increase in morbidity and mortality globally (1). The prevalence of childhood obesity and other CVD risk factors has been increasing over the past few decades in both developing and developed countries (2, 3). Globally, 39 million children under 5 years old and 340 million children and adolescents are reported to be overweight and obese (4). The main causes of these increases include physical inactivity, unhealthy diet, smoking, and sedentary behavior (5, 6). The synergistic effects of demographic change, globalization, and economic growth have resulted in more children and adolescents being exposed to these behaviors than ever before (7). CVD is now increasing steadily worldwide and has become a major public health problem of the 21st century.

CVD and underlying atherosclerosis begin in childhood. Their presence and intensity are associated with known cardiovascular risk factors (8). Middle school students are in their teenage stage, which is an important life stage. Lifestyle behaviors during this stage have the potential to carry over into higher life stages, and risk factors are more prevalent among adolescents (9, 10). A recent longitudinal study has clarified the direct link between cardiovascular risk factors in childhood and CVD in young adulthood (9), calling for early identification of risk factors. Surveys show that approximately 61% of individuals show some type of atherosclerotic lesion in the coronary arteries by the end of adolescence (11). However, CVD risk factors (such as age, gender, and genetics) are non-modifiable factors for the development of CVD. However, modifiable factors include unhealthy diet, physical inactivity, smoking, obesity, dyslipidemia, and hypertension (12). By making improvements in health approaches and behaviors in relation to identified CVD risk factors, we have the opportunity to reduce the impacts of these modifiable factors on our health, thereby reducing the risk of CVD (13). This study found that physical activity (PA) plays an important role in reducing the risk of chronic diseases, including obesity (14). Knowledge, attitudes, and practices (KAP) associated with CVD risk factors are found to be influenced by socioeconomic factors, social practices, and behavioral patterns (15). Therefore, to understand the KAP of adolescents is necessary. In addition, KAP studies are essential when evaluating interventions related to education and specifying effective measures aimed at improving the population.

This descriptive cross-sectional study was conducted on 17,731 junior high school and high school students from 50 schools in 16 provinces of China. This is the first national study in China to examine knowledge, attitudes, and practices related to CVD risk factors among middle school students. Focusing on the middle school students population in China, this study attempts to provide a framework of information to better understand the extent of our knowledge of cardiovascular health issues and to take positive health actions to increase awareness of CVD and risk factors.

## 2 Methods

### 2.1 Sampling method

**Figure 1:**
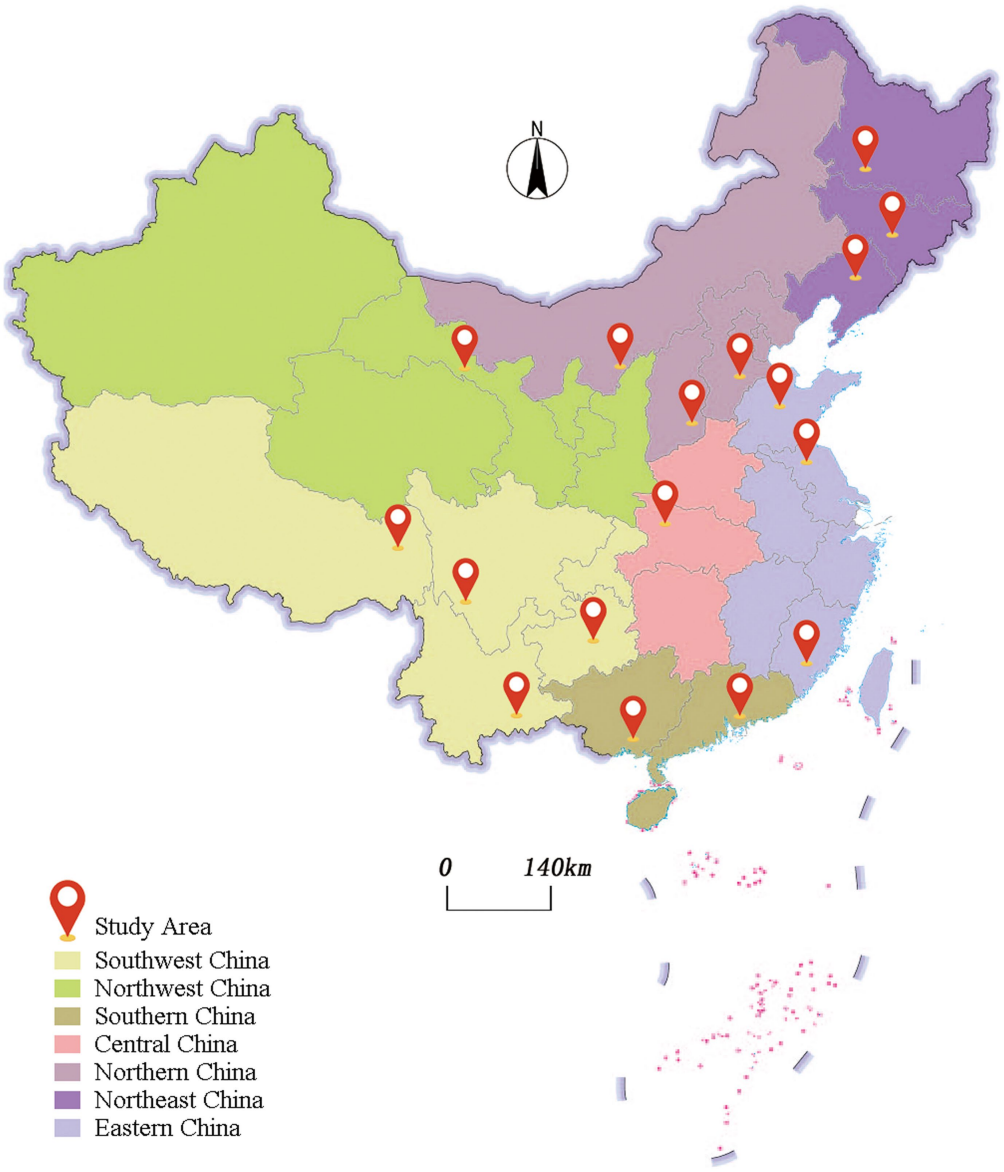
Map of the survey areas (prepared by Adobe Illustrator Artwork 23.0).

A stratified cluster random sampling method was used in this study. The seven geographic subregions of China (North China, Northeast China, East China, Central China, South China, Southwest China, and Northwest China) were used as the basis for sampling stratification. Within each geographic subregion, we selected 1–3 provinces; and 1–2 junior high schools and 1–2 high schools were selected from each province. From each school, two classes were randomly selected for each grade level. In the end, our survey encompassed 16 provinces and 50 schools (see the Figure 1).

### 2.2 The sample size calculation

Based on previous research studies on adolescents’ knowledge of chronic disease prevention, the adolescents’ knowledge rate of chronic disease prevention was 50.8% (16). With a specified tolerance error of 3% and a confidence level of 1 − *α* = 0.95, the sample size to be surveyed was calculated using the PASS 2021 software, resulting in *N* = 1,098. Considering the presence of seven stratification factors in the survey area, the sample size was adjusted to 1,098 × 7 = 7,686. Accounting for potential invalid questionnaires, the sample size was increased by 10%, resulting in a final sample size of at least 8,540.

### 2.3 Participants

We surveyed 17,731 middle school students in China using a questionnaire in June–July 2023. Of these, 8,145 (45.94%) boys and 9,586 (54.06%) girls; 7,473 (42.15%) in cities, 4,493 (25.34%) in townships, and 5,765 (32.51%) in rural areas; 13,273 (74.86%) Han Chinese and 4,458 (25.14%) ethnic minorities; 8,118 (45.78%) junior high school students; and 9,613 (54.22%) in high schools.

Before completing the questionnaire, the researcher explained the background of the study, the purpose, the principle of anonymity and confidentiality, and the precautions and obtained signed informed consent from the participants. Inclusion criteria included middle school students who were willing to participate in the study. Exclusion criteria were failure to complete the questionnaire.

### 2.4 Questionnaire design and data collection

The self-designed questionnaire “Chinese Middle School Students’ Cardiovascular Disease Prevention Questionnaire” was used, which was validated and modified by experts, with Cronbach’s *α* = 0.829, KMO = 0.887 for the knowledge part, KMO = 0.933 for the attitude part, and KMO = 0.647 for the behavior part (*p* < 0.001), with good reliability and validity. The final survey includes the following: (1) basic information about middle school students; (2) knowledge of CVD prevention: a total of 22 entries, mainly investigating middle school students knowledge of CVD prevention. Correct answers are scored as 2 points, errors or ignorance is scored as 0 points, and the full score is 44 points; (3) attitude of CVD prevention: a total of 22 entries, using the Likert 5-level scale, the options are divided into five levels, such as very little need, no need, general, need, very much need; in order to assign a score of 1, 2, 3, 4, 5, full score of 60 points; (4) behavior of CVD prevention: a total of 19 entries, in addition to the 8 sub-items in the 1st question item does not count, the remaining 11 entries have 3 or 5 options ranging from 3 or 5, accordingly divided into 3 or 5 grades, options 3 grades are assigned 1, 3, 5 points, options 5 grades are assigned 1, 2, 3, 4, 5 points, out of a total of 55 points. The 2nd, 4th–5th, 7th, 9th–10th entries are assigned points in reverse.

Questionnaires are self-filled in a centralized classroom setting, with the investigator supervising the whole process to ensure that students answer the questionnaires independently and without interference. The questionnaires are collected on-site. The data were double-entered for comparison and error checking.

### 2.5 Statistical analysis

EpiData 3.1 software is used for data entry, and IBM SPSS version 26.0 is used for statistical analysis. Measured data are described using mean scores (SD). Count data are presented as numerical values and percentages. To assess the influence of demographic characteristics on knowledge, attitude, and practice scores, we utilize the chi-square test. Logistic regression analysis is used to explore the factors impacting knowledge, attitude, and practice scores. Statistically significant is defined as a *p*-value of <0.05.

### 2.6 Ethical issues

We obtained ethical approval for this research from our Ethics Committee on 27 June 2023 (approval number 20230627001). All participants provided written informed consent. Participant names and potentially identifying information were removed to the greatest extent possible to protect anonymity.

## 3 Results

### 3.1 Demographic characteristics

In this survey of middle school students, a total of 18,500 questionnaires were distributed. Of these, 18,169 were returned, excluding 438 invalid questionnaires, and 17,731 valid questionnaires from middle school students were collected, representing an effective response rate of 95.84%.

A total of 8,145 (45.94%) boys and 9,586 (54.06%) girls were surveyed; 7,473 (42.15%) in cities, 4,493 (25.34%) in townships, and 5,765 (32.51%) in rural areas; 13,273 (74.86%) Han Chinese and 4,458 (25.14%) ethnic minorities; 8,118 (45.78%) junior high school students and 9,613 (54.22%) in high schools; 7,631 (43.04%) students living in schools and 10,100 (56.96%) not living in schools; 14,452 (81.51%) without a history of family chronic diseases and 3,279 (18.49%) with a history of family chronic diseases as detailed in Table 1.

**Table 1:** Socio-demographic characteristics of participants (*N* = 17,731).

Characteristics	Frequency
**Residence**	
City	7,473 (42.15%)
Village	4,493 (25.34%)
Countryside	5,765 (32.51%)
**Gender**	
Boys	8,145 (45.94%)
Girls	9,586 (54.06%)
**Monthly family income**	
<2000 CNY	2,432 (13.72%)
2000–3,999 CNY	5,800 (32.71%)
4,000–5,999 CNY	4,000 (22.56%)
6,000–7,999 CNY	2,442 (13.77%)
≥8,000 CNY	3,057 (17.24%)
**Nation**	
Han Chinese	13,273 (74.86%)
Ethnic minority	4,458 (25.14%)
**Grade**	
Junior high school	8,118 (45.78%)
High school	9,613 (54.22%)
**Father’s profession**	
Farmer	5,520 (31.13%)
Worker	4,608 (25.99%)
Service worker	916 (5.17%)
Private entrepreneur	3,201 (18.05%)
Civil service	734 (4.14%)
Doctor	162 (0.91%)
Teacher	354 (2.00%)
Staff	683 (3.85%)
Unemployed	595 (3.36%)
Other	958 (5.40%)
**Mother’s profession**	
Farmer	5,791 (32.66%)
Worker	2,564 (14.46%)
Service worker	1,678 (9.46%)
Private entrepreneur	2,850 (16.07%)
Civil service	417 (2.35%)
Doctor	288 (1.62%)
Teacher	635 (3.58%)
Staff	618 (3.49%)
Unemployed	2,129 (12.01%)
Other	761 (4.29%)
**Father’s education**	
Secondary	4,318 (24.35%)
Junior	7,548 (42.57%)
Senior	3,327 (18.76%)
University	2,538 (14.31%)
**Mother’s education**	
Secondary	5,701 (32.15%)
Junior	6,926 (39.06%)
Senior	2,808 (15.84%)
University	2,296 (12.95%)
**Whether living with parents**	
Living with parents	13,235 (74.64%)
Not living with parents	4,496 (25.36%)
**Whether living at the school**	
Not living at the school	10,100 (56.96%)
Living at the school	7,631 (43.04%)
**Family history of chronic diseases**	
Have	14,452 (81.51%)
None	3,279 (18.49%)

### 3.2 CVD prevention survey KAP score for middle school students

According to the actual content of the survey, (1) the knowledge section consists of 22 questions, 2 points each, totaling 44 points. (2) The attitude section consisted of 12 questions, 5 points each, with a total score of 60 points. (3) The practice section consists of 11 questions; each question is worth 5 points, and the total score is 55.

The pass line is set at 60% of the total score. The pass rate shows that the knowledge section has the lowest pass rate of 56.98%, and the behavior section has a pass rate of 89.30% as detailed in Table 2.

**Table 2:** Scoring of knowledge, attitude, and practice on CVD prevention for middle school students.

Features	Total scores	Mean scores (SD)	Passing rate
Knowledge	44	26.88 (8.12)	56.98%
Attitude	60	53.53 (7.22)	97.35%
Practice	55	39.80 (5.96)	89.30%

### 3.3 Knowledge

Definitional questions are answered correctly as known and incorrectly answered or omitted as not known. In terms of cardiovascular prevention knowledge, the items with a correct rate of less than 60% are “the concept of CVD (58.12%),” “factors affecting CVD (52.17%),” “the dangers of a high-salt diet (57.79%),” “the definition of sedentary behavior (43.96%),” “the recommended daily activity time for adolescents (34.66%),” “risk factors for CVD (23.77%),” “behaviors that cause to diabetes (33.33%),” and “behaviors that cause to hyperlipidemia (35.59%).” The lowest number of students knowing about “the dangers of being sedentary (22.92%)” and “the definition of blood pressure (BP) in adolescents (6.83%)” and the highest number of students knowing about “behaviors cause overweight and obesity (90.62%)” are detailed in Table 3.

**Table 3:** Knowledge of cardiovascular disease prevention among middle school students (*N* = 17,731).

Content of knowledge	Frequency	Content of knowledge	Frequency
Concept of CVD	10,305 (58.12%)	Influencing factors of CVD	9,250 (52.17%)
Diseases caused by smoking	14,632 (82.52%)	The dangers of a high-fat diet	15,083 (85.07%)
Benefits of whole grain medley	13,296 (74.99%)	The dangers of a high-salt diet	10,246 (57.79%)
The dangers of staying up late	14,991 (84.55%)	Definition of sedentary	7,794 (43.96%)
Diseases caused by excessive alcohol consumption	14,910 (84.09%)	Behaviors that predispose to overweight and obesity	16,067 (90.62%)
The dangers of snacks and milk tea	15,860 (89.45%)	The dangers of overweight and obesity	11,962 (67.46%)
Indicators for determining overweight and obesity	11,619 (65.53%)	The dangers of chronic anxiety and depression	14,745 (83.16%)
Risk factors for CVD	4,214 (23.77%)	Definition of blood pressure in adolescents	1,211 (6.83%)
The dangers of prolonged sedentary behavior	4,064 (22.92%)	Preventive measures for high blood pressure	13,885 (78.31%)
Behaviors that tend to cause hyperlipidemia	6,311 (35.59%)	Behaviors that predispose to diabetes	5,910 (33.33%)
Recommended daily sleeping time for middle school students	14,061 (79.30%)	Daily recommended activity times for middle school students	6,146 (34.66%)

### 3.4 Attitude

The options for middle school students’ attitudes toward CVD prevention are set up as five levels of options (e.g., very little need, no need, average, need, very much need), and the choice of need/important/severe and very much need/very important/very severe are selected as positive attitudes. The results show that middle school students have good attitudes toward CVD prevention in general. The aspects below 90% are as follows: the danger of sleep deprivation and staying up late for cardiovascular health (89.24%), the danger of anxiety and depression to cardiovascular health (83.49%), the danger of prolonged sedentary to cardiovascular health (73.55%), and the danger of overweight and obese to cardiovascular health (88.16%), attitude toward adopting a healthy lifestyle (82.14%), and receiving health education on CVD prevention (86.67%) as detailed in Table 4.

**Table 4:** Positive attitudes toward CVD prevention among middle school students (*N* = 17,731).

Content of attitude	Frequency	Percentage
CVD needs to be prevented in adolescence	16,209	91.42%
A sensible diet is important for cardiovascular health	16,508	93.10%
Physical activity is important in preventing CVD	16,281	91.82%
Smoking is dangerous for cardiovascular health	16,478	92.93%
Excessive alcohol consumption is dangerous for cardiovascular health	16,465	92.86%
Lack of sleep and late nights are dangerous for cardiovascular health	15,823	89.24%
Anxiety and depression are dangerous for cardiovascular health	14,803	83.49%
Being sedentary is dangerous for cardiovascular health	13,041	73.55%
Hypertension, hyperglycemia, and hyperlipidemia are dangerous for cardiovascular health	16,280	91.82%
Overweight and obesity are dangerous for cardiovascular health	15,632	88.16%
Attitude to adopt a healthy lifestyle	14,564	82.14%
Receive health education on cardiovascular prevention	15,368	86.67%

### 3.5 Practice

The statistical results show that 19.15% of middle school students ate sweets and sugary drinks 0 times, and 5.58% more than 7 times per week; 32.76% ate high-fat and oily food 0 times, and 3.18% more than 7 times per week; 42.91% ate salty food 0 times, and 3.20% more than 7 times per week; 13.42% ate whole grains and miscellaneous grains 0 times, and 17.61% more than 7 times per week; eating vegetables and fruits 0 times accounted for 3.47%, more than 7 times accounted for 42.80% per week; drinking dairy products 0 times accounted for 9.23%, more than 7 times accounted for 27.20% per week; eating aquatic products 0 times accounted for 34.08%, more than 7 times accounted for 5.08% per week; eating nuts 0 times accounted for 30.30%, more than 7 times accounted for 7.66% per week. Middle school students ate various types of food more centered on 1-3 times per week as detailed in Table 5.

**Table 5:** Frequency of daily meals per week among middle school students (*N* = 17,731).

Features	0 times a week	1–3 times a week	4–6 times a week	7 times a week
Sweets and sugary drinks	3,396 (19.15%)	11,567 (65.24%)	1779 (10.03%)	989 (5.58%)
High fat and oil food	5,808 (32.76%)	9,976 (56.26%)	1,383 (7.80%)	564 (3.18%)
Salty food	7,608 (42.91%)	8,312 (46.88%)	1,243 (7.01%)	568 (3.20%)
Whole grains and mixed grains	2,379 (13.42%)	8,256 (46.56%)	3,973 (22.41%)	3,123 (17.61%)
Vegetables and fruits	616 (3.47%)	4,359 (24.58%)	5,168 (29.15%)	7,588 (42.80%)
Dairy product	1,636 (9.23%)	6,784 (38.26%)	4,489 (25.32%)	4,822 (27.20%)
Aquatic product	6,042 (34.08%)	9,026 (50.91%)	1763 (9.94%)	900 (5.08%)
Nuts	5,372 (30.30%)	8,609 (48.55%)	2,392 (13.49%)	1,358 (7.66%)

The percentage of students who are not picky eaters is 54.51%. The percentages of non-smokers and non-drinkers both are over 85%. In total, 64.07% of students pay attention to their BP, blood glucose, and blood lipids. In total, 43.59% of students eat breakfast less than 7 times a week. In total, 40.27% of students have an average of more than 1 h of screen time a day. In total, 48.64% of students have a daily sleep time of less than 8 h. In total, 33.99% of students go to sleep before 12 o’clock every week. In total, 30.91% of students experience anxiety and depression 1–2 times a week. In total, 15.39% of students do not get up and move their bodies after sitting for a long time. In total, 17.61% of students do not control their diets and exercise regularly in order to maintain a healthy weight as detailed in Table 6.

**Table 6:** Daily life behaviors for CVD prevention among middle school students (*N* = 17,731).

Content of practice	Frequency
**Frequency of late bedtime (after 12 o’clock) in the last 7 days**	
0 day	6,027 (33.99%)
1–2 days	5,736 (32.35%)
3–4 days	2,624 (14.80%)
5–6 days	1,228 (6.93%)
7 days	2,116 (11.93%)
**Frequency of anxiety and depression in the past 7 days**	
None	7,309 (41.22%)
1–2 times	5,480 (30.91%)
3–4 times	2,775 (15.65%)
5–6 times	924 (5.21%)
7 times	1,243 (7.01%)
**Do you concern your blood pressure, blood sugar, and lipids**	
No concern	6,371 (35.93%)
Concern	8,176 (46.11%)
Very much concern	3,184 (17.96%)
**Do you get up and move your body after being sedentary?**	
No	2,728 (15.39%)
Sometimes	9,135 (51.52%)
Always	5,868 (33.09%)
**Frequency of smoking**	
Non-smoking	16,849 (95.02%)
Sometimes	512 (2.89%)
Always	370 (2.09%)
**Frequency of drinking**	
Not drinking	15,602 (87.99%)
Sometimes	1814 (10.23%)
Always	315 (1.78%)
**Average screen time per day over the past 7 days**	
<30 min	5,557 (31.34%)
30–60 min	5,033 (28.39%)
≥1 h	7,141 (40.27%)
**Daily controlled diet and regular exercise to maintain a healthy weight**	
No	3,123 (17.61%)
Sometimes	9,650 (54.42%)
Always	4,958 (27.96%)
**Daily sleep duration in the last 7 days**	
<8 h	8,624 (48.64%)
8–10 h	7,866 (44.36%)
≥10 h	1,241 (7.00%)
**Frequency of breakfast in the last 7 days**	
0 day	1,362 (7.68%)
1–2 days	1,493 (8.42%)
3–4 days	2,198 (12.40%)
5–6 days	2,676 (15.09%)
7 days	10,002 (56.41%)
**Are you a picky (not vegetables) eater?**	
Not picky	9,666 (54.51%)
Not eating one or two vegetables	5,766 (32.52%)
Not eating more than three vegetables	2,299 (12.97%)

### 3.6 Univariate analysis of KAP for CVD prevention among middle school students

Statistically significant differences are observed in terms of residence, gender, grade, co-residence with parents, parental occupation, parental education, ethnicity, monthly family income, and family history of chronic diseases about middle school students with good knowledge scores on CVD prevention. Knowledge is better among middle school students residing in urban (62.80%, *n* = 4,693) than in township (53.31%, *n* = 2,395) and rural (52.30%, *n* = 3,015). Girls (60.38%, *n* = 5,788) have better knowledge than boys (52.98%, *n* = 4,315). High school students (64.91%, *n* = 6,240) have better rates of good knowledge than junior high school students (47.59%, *n* = 3,863). The rate of good knowledge of middle school students increased with the higher level of parental education as detailed in Table 7 and Supplementary Table S1.

**Table 7:** Chi-square test of KAP for CVD prevention middle school students.

Features	Knowledge		Attitude		Practice	
	*ꭓ* ^2^	*p*-value	*ꭓ* ^2^	*p*-value	*ꭓ* ^2^	*p*-value
Residence	179.542	<0.001	111.386	<0.001	34.211	<0.001
Gender	98.435	<0.001	1.341	0.247	39.722	<0.001
Grade	539.015	<0.001	2.657	0.103	1210.354	<0.001
Whether living at the school	1.495	0.221	29.588	<0.001	11.929	0.001
Whether living with parents	27.274	<0.001	24.123	<0.001	59.755	<0.001
Father’s profession	195.520	<0.001	124.773	<0.001	24.718	0.003
Mother’s profession	176.581	<0.001	105.606	<0.001	19.507	0.021
Father’s education	303.558	<0.001	176.211	<0.001	21.424	<0.001
Mother’s education	227.071	<0.001	97.204	<0.001	30.748	<0.001
Nation	85.935	<0.001	1.238	0.266	14.242	<0.001
Monthly family income	178.057	<0.001	23.515	<0.001	4.111	0.391
Family history of chronic diseases	14.996	<0.001	5.537	0.018	70.161	<0.001

Good attitude scores on CVD prevention among middle school students are statistically significant, which is observed in terms of residence, whether lives in school, co-residence with parents, parental occupation, parental education, monthly family income, and family history of chronic diseases. Middle school students living in cities (62.02%, *n* = 4,635) have better attitudes than those living in towns (55.46%, *n* = 2,492) and rural areas (53.32%, *n* = 3,074). Middle school students living with parents (58.59%, *n* = 7,755) have better attitudes than those living with other relatives (54.40%, *n* = 2,446) as detailed in Table 7 and Supplementary Table S1.

Good practices in CVD prevention among middle school students are statistically significant, which is observed in terms of residence, gender, grade, whether lives in school, co-residence with parents, parental occupation, parental education, ethnicity, and family history of chronic diseases. Middle school students living in townships (54.13%, *n* = 2,432) and rural areas (53.86%, *n* = 3,105) have better good behavior than those living in urban (49.54%, *n* = 3,702). Boys (54.67%, *n* = 4,453) have better behavior than girls (49.93%, *n* = 4,786). Junior high school students (66.31%, *n* = 5,383) have better behavior than high school students (40.11%, *n* = 3,856) as detailed in Table 7 and Supplementary Table S1.

### 3.7 Logistic regression of factors influencing KAP for CVD prevention in middle school students

Levels of CVD prevention knowledge, attitudes, and practices are used as the dependent variable. The statistically significant factors identified through Chi-square test are used as the independent variables.

Logistic regression results show that residence, grade, gender, whether living with parents, mother’s occupation, parental education, monthly family income, ethnicity, and family history of chronic disease are influencing factors of middle school students CVD prevention knowledge scores. Middle school students living in townships (95% CI: 0.757, 0.893; *p* < 0.001) are more likely to have poorer knowledge than those living in cities. High school students (95% CI: 1.920, 2.175; *p* < 0.001) are more likely to have better knowledge than junior high school students. Girls (95% CI: 1.314, 1.487; *p* < 0.001) are more likely to have good knowledge than boys. Middle school students living with other relatives (95% CI: 0.811, 0.936; *p* < 0.001) are more likely to have poor knowledge than those living with both parents. Students whose father’s education is junior high school (95% CI: 1.171, 1.388; *p* < 0.001), senior high school (95% CI: 1.296, 1.623; *p* < 0.001), and university (95% CI: 1.302, 1.757; *p* < 0.001) are more likely to have better knowledge than students whose fathers are primary school students. Middle school students whose mothers’ education is high school (95% CI: 1.085, 1.370; *p* < 0.05) and university (95% CI: 1.082, 1.466; *p* < 0.05) are more likely to have better knowledge than students whose mothers are in primary school. The higher the monthly family income, the more likely students are to have better knowledge. Students from ethnic minorities (95% CI: 0.693, 0.803; *p* < 0.001) are more likely to have poorer knowledge than Han Chinese. Students without a family history of chronic disease (95% CI: 0.852, 0.987; *p* < 0.001) are more likely to have poorer knowledge than students with a family history of chronic disease.

Living in an urban area, not living in school, living with parents, father’s occupation as a teacher, father’s higher education level, and having a family history of chronic diseases are the influencing factors of better attitude among middle school students. Living in towns and villages, boys, junior high school, living with parents, higher educational level of parents, and no family history of chronic diseases are the influencing factors of better behaviors as detailed in Supplementary Table S2.

## 4 Discussion

As the prevalence of CVD and risk factors is rapidly increasing globally, primary prevention, early diagnosis, and educational preventive measures are now prioritized (17). The aim of this study is to understand the current status of knowledge, attitudes, and practices related to CVD prevention among middle school students. This survey shows that low awareness of CVD prevention among Chinese middle school students is a major health problem. Understanding CVD can improve middle school students’ awareness of the early dangers of CVD, which can ultimately reduce the life-threatening consequences associated with the disease.

Our results showed that awareness rate of knowledge of the Chinese middle school students’ CVD prevention survey was 56.98%. The result was higher than Sitaula D et al. survey on diabetes and hypertension among school students in Nepal (18). Because there had hardly been any regular school-based awareness campaigns or health intervention programs focusing on NCDs such as diabetes and hypertension.

It was found that students’ knowledge of “concept of CVD (58.12%)” and “factors affecting CVD” (52.17%) was inadequate, which may be due to the fact that students did not receive adequate education on the importance and risk factors of CVD (19). In addition, the knowledge of “behaviors causing overweight and obesity (90.62%)” and “hazards of snacks and milk tea (89.45%)” was better, but there was insufficient knowledge of “hazards of prolonged sedentary behavior (22.92%)” and “definition of BP in adolescents (6.83%)” probably because of the imbalance in the transmission of health information. The hazards of overweight and obesity and snacks and milk tea were more prominent in health education and publicity. The definition of prolonged sedentary behavior and BP in adolescents might not have received enough attention (16).

In our study, 91.82% of students had a positive attitude toward PA being important in the prevention of CVD, but 73.55% had a positive attitude toward sedentary behavior being harmful to cardiovascular health, which was the lowest. The concepts of sedentary behavior and lack of PA are not the same, and meeting PA recommendations does not guarantee that one will not be sedentary (20). Thus, they are independent modifiable risk factors for CVD (21). Promoting PA and reducing sedentary behavior are strategies to prevent CVD. Light or moderate PA as a substitute for sedentary behavior has been found to reduce type 2 diabetes, cardiovascular mortality, and all-cause mortality (22, 23). In total, 82.14% of students indicated that they were willing to adopt a healthy lifestyle and 86.67% were willing to receive health education on CVD prevention. It shows that these students are aware of health issues and the importance of cardiovascular health. Students are willing to take positive actions to improve their lifestyles and improve their health. Therefore, schools and the community can take measures to improve students’ awareness of health issues and encourage them to adopt healthy behaviors. However, there are still some students who are reluctant to adopt a healthy lifestyle and unwilling to receive health education on CVD prevention. We should fully understand the possible reasons, change their attitudes, and improve their awareness of the dangers of CVD.

In total, 7.68% of students did not eat breakfast every day, which is similar to the findings by Bassi et al. (24). In total, 56.41% of students eat breakfast every day. A cross-sectional survey showed urban students prioritized having a healthy diet and ate daily breakfast compared to rural (25). It is possible that the economy in rural areas is not as stable as that of students in urban areas, which makes it difficult for families of rural students to provide an adequate breakfast. A study confirmed that eating breakfast away from home is 1.7 times more likely to lead to obesity than eating breakfast at home (26). This may be due to the rapid development of the food service industry, which has led to an increase in the number of students eating out. Other dietary factors included vegetables and fruits, which were not eaten daily by only 3.47% of students and 1–3 times a week by 24.58% of students. The study of Indian adolescents reported that 11.3% of the students did not eat fruits daily (24). In a comparison of 49 low- and middle-income countries, Indian adolescents had the highest proportion of fruit and vegetable consumption in line with WHO recommendations (27). A survey on type II diabetes in American adolescents revealed that participants consumed an average of 1.5 snacks per day. Specifically, 23.0% reported consuming two snacks, 5.1% consuming three snacks. Moreover, the survey observed that these adolescents spend 1.9 times per week on fast food, with 49.3% of them reporting twice-weekly fast-food consumption, and 24.3% three times per week (28). These findings highlighted the prevalence of snacks and fast-food consumption among the adolescent population. In addition, we found that 64.07% of students are concerned about their BP, blood glucose, and blood lipids, which is higher than Asante et al. (29). Regular BP, blood glucose, and lipid testing are essential for early prevention and detection of hypertension and diabetes. Studies have found that the risk of CVD, including hypertension and diabetes, increases with age (30). Our study showed that 84.61% of students would get up and move their bodies after being sedentary. With the development of electronic technology, the sedentary time of students watching TV, reading mobile phones, and surfing the Internet has been increasing, and outdoor activities have been decreasing. This lifestyle will increase the risk of obesity and CVD in teenagers (31). Our study found that 82.39% of students would maintain a healthy weight by controlling their diet and exercising regularly, which is in line with other survey results (32, 33).

Our study found that students living in urban areas had higher knowledge scores than those living in towns and rural areas because probably urban areas usually have richer educational resources and higher quality of education, which would improve the learning performance of urban students and broaden their knowledge domains. However, the proportion of students living in towns and villages with good behavior, was higher than for those living in cities because possibly towns and villages often have healthier lifestyles, such as healthier diets and increased PA, which can help to reduce the risk of CVD. This was confirmed by a study in Bangladesh (34). Girls scored higher knowledge scores than boys. It is possible that there is a difference in the importance attached to health by students of different genders. However, the percentage of girls scoring good behaviors was lower than that of boys, probably because girls were more physically inactive and consumed more snacks. Gender is an important social determinant of health behaviors from childhood to adolescence to adulthood, and boys will have significantly higher levels of PA than girls during their formative years (35, 36). This inter-gender gap increases during the adolescent transition. The study showed that the percentage of high school students with good knowledge scores was higher than that of junior high school students. However, the percentage of high school students with good practice scores was lower than that of junior high school students, probably due to the pressure of high school, lack of PA, and reduced sleep. A survey from Poland on obesity and nutritional knowledge among adolescents showed that nutritional knowledge among adolescents was positively associated with adhering to a healthy lifestyle (37). Furthermore, adolescents with higher nutritional knowledge exhibited lower trends for fast-food consumption and sedentary behavior. This result emphasized the significant influence of knowledge levels on individual lifestyle choices, particularly in the adolescent population. Understanding of health knowledge may make adolescents more inclined to make healthful lifestyle choices.

Students living with both parents with good knowledge scores and good behaviors were higher than those living with other relatives, probably due to the fact that students living with both parents had easier access to family support and a more favorable educational environment, which helped to improve students’ motivation and learning standards (38). The knowledge scores of students whose parents’ occupations were teachers and doctors were significantly higher than those of the other groups, which may be due to the fact that parents with higher socioeconomic status were more likely to pass on knowledge about CVD prevention to their children. Thus, their children may have acquired more relevant knowledge. In addition, more educated parents were more likely to provide educational support and resources to facilitate their children’s health education, which is consistent with the findings of Ishikawa et al. (39). This suggests that parental occupation and educational level have a significant impact on students’ knowledge of CVD prevention, formation of awareness, and development of behaviors. A cross-sectional survey in Germany found a relationship between parental health literacy and certain health behaviors in children. Children whose parents had higher health literacy were found to have higher rates of consumption of vegetables and salads and engaged in more physical activity than their counterparts (40). This finding highlighted the positive impact of parental health literacy on children’s dietary and exercise habits. Our survey found students in Han Chinese have higher knowledge scores than students in ethnic minority may be due to the fact that in some areas, educational resources are more concentrated in areas inhabited by Han Chinese, which contributes to higher knowledge scores for Han Chinese students. Moreover, there may be cultural and social differences among different ethnicities, which may affect students’ attitudes and behaviors (41). There are differences in the knowledge, attitudes and practices scores of middle school students with different monthly family incomes. The results of the study are consistent with the results of Akter et al. (15). This may be due to the fact that families of different economic levels have different importance on health knowledge, which leads to differences in the students’ acceptance of health education and mass communication.

KAP related to CVD prevention was found to be influenced by socioeconomic factors and social behavior patterns (15). Our study found that girls were more likely to have good levels of CVD knowledge, with the opposite result being that girls were more likely to have poorer behaviors, while boys were more likely to have better behaviors. Similar studies have also shown better behavioral scores for boys compared to better attitude scores for girls (42). Previous studies have shown that educational level is associated with awareness of hypertension and diabetes. Educational level may influence health in terms of health knowledge, healthcare competencies, behaviors, and psychosocial aspects (43). This also supports some of the research (44). In addition, a family history of chronic disease affected the knowledge, which is consistent with the findings of Niermann et al. (45). This may be due to the fact that people with a family history of chronic disease are more likely to pay attention to knowledge and their own health status than the general population (46). Students living with both parents showed better knowledge, attitudes, and behaviors on CVD prevention. A study showed that students living with both parents had higher self-rated health scores and were less likely to experience mental health issues (47). This may imply that the home environment and family education play an important role in educating students about cardiovascular health.

This study is the first study of CVD prevention on knowledge, attitudes, and practices among middle school students in China at present. It mainly focuses on junior high school and high school students. Effectively representing all geographic subregions of China (central, northern, eastern, southern, northwestern, southwestern, and northeastern). It also encompasses urban, township, and rural areas, with an inclusive approach that extends to ethnic minority regions.

However, there are some limitations to this study. Despite the comprehensive coverage of seven major geographical subregions in China and participants from diverse socioeconomic backgrounds, certain areas may not have been included in the investigation due to resource constraints. Additionally, due to the lack of a standardized questionnaire on KAP related to CVD, we utilized a self-administered questionnaire that has undergone rigorous reliability and validity testing. The data collection relies on students’ self-reports, so the data collected may have been influenced by recall bias.

## 5 Conclusion

This study revealed poorer levels of CVD prevention knowledge among Chinese middle school students, most of whom lacked knowledge of CVD concepts and associated risk factors, but displayed better attitudes and practices. Moreover, influenced by socio-demographic, socioeconomic, and socio-behavioral patterns, we observed that those living in urban areas, high school students, girls, those living with parents, those whose parents had a higher education level, those who came from higher family income, Han Chinese, and those having a family history of chronic disease were more likely to have a good level of CVD knowledge. We should take effective measures to strengthen cardiovascular health education for students, improve their health knowledge, and help them develop correct health attitudes and adopt positive health practices.

## Data availability statement

The raw data supporting the conclusions of this article will be made available by the authors, without undue reservation.

## Ethics statement

The studies involving humans were approved by the Human Ethics Committee of Guangxi Normal University. The studies were conducted in accordance with the local legislation and institutional requirements. Written informed consent for participation in this study was provided by the participants’ legal guardians/next of kin.

## Author contributions

XY: Data curation, Formal analysis, Investigation, Methodology, Project administration, Supervision, Writing – original draft, Writing – review & editing. QQ: Data curation, Formal analysis, Investigation, Methodology, Project administration, Supervision, Writing – original draft, Writing – review & editing. YW: Data curation, Investigation, Supervision, Writing – review & editing. ZM: Investigation, Writing – review & editing. QL: Investigation, Writing – review & editing. FZ: Investigation, Writing – review & editing. YH: Investigation, Writing – review & editing. HW: Funding acquisition, Project administration, Resources, Supervision, Writing – original draft, Writing – review & editing, Investigation.
