# Supplementary material for: Knowledge, attitudes, and practices regarding cardiovascular disease prevention among middle school students in China: a cross-sectional study
Source: Front Public Health. 2024 Jan 26;12:1301829. doi: 10.3389/fpubh.2024.1301829 (PMC10853328; doi:10.3389/fpubh.2024.1301829)
Supplement: Supplementary file 1 [file Data_Sheet_1.docx]

Supplementary Material

# Supplementary Tables 1: Good KAP in CVD prevention among middle school students (N=17731)

# Supplementary Tables 2: Results of binary logistic regression of KAP on CVD prevention among secondary school students

| **Supplementary table 1** Good KAP in CVD prevention among middle school students (N=17731) | | | | |
| --- | --- | --- | --- | --- |
| **Features** | **Frequency** | **Good knowledge** | **Good attitude** | **Good practice** |
| **Residence** |  |  |  |  |
| City | 7473 | 4693(62.80%) | 4635(62.02%) | 3702(49.54%) |
| Village | 4493 | 2395(53.31%) | 2492(55.46%) | 2432(54.13%) |
| Countryside | 5765 | 3015(52.30%) | 3074(53.32%) | 3105(53.86%) |
| **Gender** |  |  |  |  |
| Boys | 8145 | 4315(52.98%) | 4648(57.07%) | 4453(54.67%) |
| Girls | 9586 | 5788(60.38%) | 5553(57.93%) | 4786(49.93%) |
| **Grade** |  |  |  |  |
| Junior school | 8118 | 3863(47.59%) | 4617(56.87%) | 5383(66.31%) |
| High school | 9613 | 6240(64.91%) | 5584(58.09%) | 3856(40.11%) |
| **Whether living at the school** |  |  |  |  |
| Not Living at the school | 10100 | 5715(56.58%) | 5988(59.29%) | 5149(50.98%) |
| Living at the school | 7631 | 4388(57.50%) | 4213(55.21%) | 4090(53.60%) |
| **Whether living with parents** |  |  |  |  |
| Living with parents | 13235 | 7691(58.11%) | 7755(58.59%) | 7120(53.80%) |
| Not living with parents | 4496 | 2412(53.65%) | 2446(54.40%) | 2119(47.13%) |
| **Father’s profession** |  |  |  |  |
| Farmer | 5520 | 2817(51.03%) | 2982(54.02%) | 2974(53.88%) |
| worker | 4608 | 2614(56.73%) | 2539(55.10%) | 2377(51.58%) |
| Service worker | 916 | 556(60.70%) | 566(61.79%) | 490(53.49%) |
| Private entrepreneur | 3201 | 1956(61.11%) | 1917(59.89%) | 1626(50.80%) |
| Civil service | 734 | 498(67.85%) | 476(64.85%) | 350(47.68%) |
| Doctor | 162 | 122(75.31%) | 101(62.35%) | 87(53.70%) |
| Teacher | 354 | 234(66.10%) | 266(75.14%) | 194(54.80%) |
| Staff | 683 | 438(64.13%) | 423(61.93%) | 376(55.05%) |
| Unemployed | 595 | 351(58.99%) | 355(59.66%) | 294(49.41%) |
| Other | 958 | 517(53.97%) | 576(60.13%) | 471(49.16%) |
| **Mather’s profession** |  |  |  |  |
| Farmer | 5791 | 2977(51.41%) | 3122(53.91%) | 3115(53.79%) |
| worker | 2564 | 1466(57.18%) | 1411(55.03%) | 1328(51.79%) |
| Service worker | 1678 | 1038(61.86%) | 1008(60.07%) | 843(50.24%) |
| Private entrepreneur | 2850 | 1714(60.14%) | 1707(59.89%) | 1460(51.23%) |
| Civil service | 417 | 283(67.87%) | 263(63.07%) | 208(49.88%) |
| Doctor | 288 | 203(70.49%) | 192(66.67%) | 153(53.13%) |
| Teacher | 635 | 391(61.57%) | 438(68.98%) | 347(54.65%) |
| Staff | 618 | 389(62.94%) | 386(62.46%) | 337(54.53%) |
| Unemployed | 2129 | 1261(59.23%) | 1218(57.21%) | 1078(50.63%) |
| Other | 761 | 381(50.07%) | 456(59.92%) | 370(48.62%) |
| **Father’s education** |  |  |  |  |
| Secondary | 4318 | 2057(47.64%) | 2167(50.19%) | 2119(49.07%) |
| Junior | 7548 | 4249(56.29%) | 4335(57.43%) | 3994(52.91%) |
| Senior | 3327 | 2115(63.57%) | 2048(61.56%) | 1765(53.05%) |
| University | 2538 | 1682(66.27%) | 1651(65.05%) | 1361(53.62%) |
| **Mather’s education** |  |  |  |  |
| Secondary | 5701 | 2885(50.61%) | 3036(53.25%) | 2807(49.24%) |
| Junior | 6926 | 3906(56.40%) | 3973(57.36%) | 3743(54.04%) |
| Senior | 2808 | 1802(64.17%) | 1731(61.65%) | 1463(52.10%) |
| University | 2296 | 1510(65.77%) | 1461(63.63%) | 1226(53.40%) |
| **Nation** |  |  |  |  |
| Han Chinese | 13273 | 7828(58.98%) | 7668(57.77%) | 7025(52.93%) |
| Ethnic minority | 4458 | 2275(51.03%) | 2533(56.82%) | 2214(49.66%) |
| **Monthly family income** |  |  |  |  |
| <2000CNY | 2432 | 1159(47.66%) | 1325(54.48%) | 1265(52.01%) |
| 2000-3999CNY | 5800 | 3155(54.40%) | 3295(56.81%) | 3012(51.93%) |
| 4000-5999CNY | 4000 | 2348(58.70%) | 2290(57.25%) | 2066(51.65%) |
| 6000-7999CNY | 2442 | 1546(63.31%) | 1478(60.52%) | 1318(53.97%) |
| ≥8000CNY | 3057 | 1895(61.99%) | 1813(59.31%) | 1578(51.62%) |
| **Family history of chronic diseases** | |  |  |  |
| Have | 4147 | 2471(59.59%) | 2452(59.13%) | 1925(46.42%) |
| None | 13584 | 7632(56.18%) | 7749(57.05%) | 7314(53.84%) |

| **Supplementary table 2** Results of binary logistic regression of KAP on CVD prevention among secondary school students | | | | | | | | | | | |  |
| --- | --- | --- | --- | --- | --- | --- | --- | --- | --- | --- | --- | --- |
| **Features** | **Knowledge** | | |  | **Attitude** | | |  | **Practice** | | |  |
|  | **β(SE)** | **OR (95%CI)** | **P-value** |  | **β(SE)** | **OR (95%CI)** | **P-value** |  | **β(SE)** | **OR (95%CI)** | **P-value** | |
| **Residence** |  |  | <0.001 |  |  |  | <0.001 |  |  |  | <0.001 | |
| City* |  |  |  |  |  |  |  |  |  |  |  | |
| Village | -0.20(0.04) | 0.82(0.76, 0.89) | <0.001 |  | -0.18(0.04) | 0.84(0.77,0.91) | <0.001 |  | 0.13(0.04) | 1.14(1.05,1.24) | 0.002 | |
| Countryside | -0.10(0.05) | 0.91(0.83, 0.99) | 0.036 |  | -0.19(0.05) | 0.83(0.76,0.90) | <0.001 |  | 0.18(0.05) | 1.20(1.09,1.31) | <0.001 | |
| **Grade** |  |  |  |  |  |  |  |  |  |  |  | |
| Junior school* |  |  |  |  |  |  |  |  |  |  |  | |
| High school | 0.71(0.03) | 2.04(1.92, 2.18) | <0.001 |  | - | - | - |  | -1.06(0.03) | 0.35(0.32,0.37) | <0.001 | |
| **Gender** |  |  |  |  |  |  |  |  |  |  |  | |
| Boys* |  |  |  |  |  |  |  |  |  |  |  | |
| Girls | 0.34(0.03) | 1.40(1.31, 1.49) | <0.001 |  | - | - | - |  | -0.17(0.03) | 0.85(0.80,0.90) | <0.001 | |
| **Whether living at the school** |  |  |  |  |  |  |  |  |  |  |  | |
| Not Living at the school* |  |  |  |  |  |  |  |  |  |  |  | |
| Living at the school | - | - | - |  | -0.23(0.03) | 0.80(0.75,0.85) | <0.001 |  | 0.02(0.03) | 1.02(0.96,1.09) | 0.578 | |
| **Whether living with parents** |  |  |  |  |  |  |  |  |  |  |  | |
| Living with parents* |  |  |  |  |  |  |  |  |  |  |  | |
| Not living with parents | -0.14(0.04) | 0.87(0.81, 0.94) | <0.001 |  | -0.15(0.04) | 0.86(0.80,0.92) | <0.001 |  | -0.27(0.04) | 0.76(0.71,0.82) | <0.001 | |
| **Father’s profession** |  |  | 0.391 |  |  |  | 0.012 |  |  |  | 0.576 | |
| Farmer* |  |  |  |  |  |  |  |  |  |  |  | |
| worker | 0.06(0.06) | 1.06(0.94,1.20) | 0.306 |  | -0.04(0.06) | 0.96(0.86,1.08) | 0.536 |  | -0.10(0.06) | 0.91(0.81,1.03) | 0.118 | |
| Service worker | 0.10(0.09) | 1.11(0.92,1.33) | 0.262 |  | 0.12(0.09) | 1.12(0.94,1.34) | 0.207 |  | -0.01(0.09) | 0.99(0.82,1.18) | 0.881 | |
| Private entrepreneur | 0.10(0.08) | 1.10(0.95,1.28) | 0.190 |  | 0.03(0.07) | 1.03(0.89,1.19) | 0.694 |  | -0.11(0.08) | 0.90(0.77,1.04) | 0.144 | |
| Civil service | 0.18(0.11) | 1.20(0.97,1.50) | 0.097 |  | 0.11(0.11) | 1.12(0.90,1.38) | 0.310 |  | -0.22(0.11) | 0.80(0.65,0.99) | 0.038 | |
| Doctor | 0.50(0.20) | 1.65(1.11,2.45) | 0.012 |  | -0.02(0.18) | 0.98(0.69,1.40) | 0.928 |  | 0.00(0.18) | 1.00(0.70,1.42) | 0.985 | |
| Teacher | 0.16(0.14) | 1.17(0.89,1.54) | 0.271 |  | 0.54(0.15) | 1.72(1.29,2.29) | <0.001 |  | -0.01(0.14) | 0.99(0.76,1.30) | 0.967 | |
| Staff | 0.18(0.11) | 1.20(0.96,1.49) | 0.112 |  | 0.00(0.11) | 1.00(0.81,1.24) | 0.997 |  | -0.12(0.11) | 0.88(0.71,1.10) | 0.260 | |
| Unemployed | 0.16(0.10) | 1.17(0.95,1.43) | 0.133 |  | 0.09(0.10) | 1.10(0.90,1.34) | 0.375 |  | -0.04(0.10) | 0.96(0.79,1.18) | 0.723 | |
| Other | 0.03(0.09) | 1.03(0.86,1.23) | 0.781 |  | 0.07(0.09) | 1.07(0.90,1.28) | 0.450 |  | -0.07(0.09) | 0.94(0.78,1.12) | 0.473 | |
| **Mather’s profession** |  |  | 0.011 |  |  |  | 0.289 |  |  |  | 0.483 | |
| Farmer* |  |  |  |  |  |  |  |  |  |  |  | |
| worker | 0.03(0.07) | 1.03(0.90,1.17) | 0.688 |  | 0.01(0.07) | 1.01(0.89,1.15) | 0.908 |  | 0.02(0.07) | 1.02(0.89,1.17) | 0.761 | |
| Service worker | 0.06(0.08) | 1.07(0.91,1.24) | 0.414 |  | 0.05(0.08) | 1.05(0.90,1.22) | 0.538 |  | -0.14(0.08) | 0.87(0.75,1.02) | 0.083 | |
| Private entrepreneur | -0.10(0.08) | 0.91(0.78,1.06) | 0.210 |  | 0.04(0.08) | 1.04(0.90,1.20) | 0.607 |  | -0.04(0.08) | 0.96(0.83,1.12) | 0.584 | |
| Civil service | -0.03(0.13) | 0.97(0.74,1.26) | 0.809 |  | 0.02(0.13) | 1.02(0.79,1.32) | 0.860 |  | -0.03(0.13) | 0.97(0.76,1.26) | 0.838 | |
| Doctor | 0.19(0.15) | 1.21(0.90,1.62) | 0.205 |  | 0.27(0.14) | 1.31(0.98,1.73) | 0.065 |  | -0.06(0.14) | 0.94(0.71,1.24) | 0.659 | |
| Teacher | -0.21(0.11) | 0.81(0.65,1.01) | 0.062 |  | 0.27(0.12) | 1.31(1.05,1.64) | 0.019 |  | -0.03(0.11) | 0.97(0.78,1.22) | 0.806 | |
| Staff | -0.14(0.11) | 0.87(0.70,1.09) | 0.232 |  | 0.09(0.11) | 1.09(0.88,1.36) | 0.423 |  | 0.02(0.11) | 1.02(0.82,1.28) | 0.843 | |
| Unemployed | -0.02(0.07) | 0.98(0.85,1.12) | 0.740 |  | -0.02(0.07) | 0.98(0.85,1.12) | 0.754 |  | -0.08(0.07) | 0.92(0.80,1.06) | 0.255 | |
| Other | -0.27(0.10) | 0.77(0.63, 0.93) | 0.008 |  | 0.09(0.10) | 1.09(0.90,1.32) | 0.385 |  | -0.16(0.10) | 0.85(0.70,1.04) | 0.111 | |
| **Father’s education** |  |  | <0.001 |  |  |  | <0.001 |  |  |  | <0.001 | |
| Secondary* |  |  |  |  |  |  |  |  |  |  |  | |
| Junior | 0.24(0.04) | 1.28(1.17, 1.39) | <0.001 |  | 0.26(0.04) | 1.30(1.19,1.41) | <0.001 |  | 0.13(0.04) | 1.14(1.04,1.24) | 0.003 | |
| Senior | 0.37(0.06) | 1.45(1.30, 1.62) | <0.001 |  | 0.37(0.06) | 1.45(1.30,1.62) | <0.001 |  | 0.25(0.06) | 1.29(1.15,1.44) | <0.001 | |
| University | 0.41(0.08) | 1.51(1.30, 1.76) | <0.001 |  | 0.42(0.08) | 1.53(1.32,1.77) | <0.001 |  | 0.26(0.08) | 1.30(1.12,1.50) | 0.001 | |
| **Mather’s education** |  |  | 0.003 |  |  |  | 0.554 |  |  |  | 0.008 | |
| Secondary* |  |  |  |  |  |  |  |  |  |  |  | |
| Junior | 0.06(0.04) | 1.07(0.98, 1.16) | 0.127 |  | 0.02(0.04) | 1.02(0.94,1.11) | 0.596 |  | 0.15(0.04) | 1.16(1.06,1.26) | 0.001 | |
| Senior | 0.20(0.06) | 1.22(1.09, 1.37) | 0.001 |  | 0.06(0.06) | 1.06(0.95,1.19) | 0.312 |  | 0.12(0.06) | 1.13(1.01,1.27) | 0.036 | |
| University | 0.23(0.08) | 1.26(1.08, 1.47) | 0.003 |  | -0.03(0.08) | 0.97(0.84,1.13) | 0.705 |  | 0.11(0.08) | 1.12(0.97,1.30) | 0.137 | |
| **Monthly family income** |  |  | <0.001 |  |  |  | 0.070 |  |  |  |  | |
| <2000CNY* |  |  |  |  |  |  |  |  |  |  |  | |
| 2000-3999CNY | 0.12(0.05) | 1.12(1.02, 1.24) | 0.021 |  | 0.01(0.05) | 1.01(0.91,1.11) | 0.909 |  | - | - | - | |
| 4000-5999CNY | 0.18(0.06) | 1.20(1.07, 1.34) | 0.002 |  | -0.05(0.06) | 0.95(0.85,1.06) | 0.330 |  | - | - | - | |
| 6000-7999CNY | 0.30(0.07) | 1.35(1.19, 1.54) | <0.001 |  | 0.00(0.06) | 1.00(0.88,1.13) | 0.966 |  | - | - | - | |
| ≥8000CNY | 0.17(0.06) | 1.19(1.04, 1.35) | 0.009 |  | -0.13(0.06) | 0.88(0.78,0.99) | 0.039 |  | - | - | - | |
| **Nation** |  |  |  |  |  |  |  |  |  |  |  | |
| Han Chinese* |  |  |  |  |  |  |  |  |  |  |  | |
| Ethnic minority | -0.29(0.04) | 0.75(0.69, 0.80) | <0.001 |  | - | - | - |  | -0.05(0.04) | 0.95(0.88,1.03) | 0.191 | |
| **Family history of chronic diseases** | |  |  |  |  |  |  |  |  |  |  | |
| Have* |  |  |  |  |  |  |  |  |  |  |  | |
| None | -0.09(0.04) | 0.92(0.85, 0.99) | 0.021 |  | -0.07(0.04) | 0.93(0.86,1.00) | 0.044 |  | 0.30(0.04) | 1.35(1.25,1.45) | <0.001 | |
| *As a reference |  |  |  |  |  |  |  |  |  |  |  | |
